# Supplementary material for: Multi‐dataset identification of innovative feature genes and molecular mechanisms in keratoconus
Source: J Cell Mol Med. 2024 Sep 19;28(18):e70079. doi: 10.1111/jcmm.70079 (PMC11412914; doi:10.1111/jcmm.70079)
Supplement: Supplementary file 2 — Material S2. [file JCMM-28-e70079-s002.zip › 7.GSEA analysis/1.ARL11/KEGG_GNRH_SIGNALING_PATHWAY.html]

Details for gene set KEGG\_GNRH\_SIGNALING\_PATHWAY[GSEA]

|  || Dataset | ARL11.group.cls#h\_versus\_l |
| Phenotype | group.cls#h\_versus\_l |
| Upregulated in class | h |
| GeneSet | KEGG\_GNRH\_SIGNALING\_PATHWAY |
| Enrichment Score (ES) | 0.18478857 |
| Normalized Enrichment Score (NES) | 0.8388366 |
| Nominal p-value | 0.76506025 |
| FDR q-value | 1.0 |
| FWER p-Value | 1.0 |
Table: GSEA Results Summary

  

Fig 1: Enrichment plot: KEGG\_GNRH\_SIGNALING\_PATHWAY      
 Profile of the Running ES Score & Positions of GeneSet Members on the Rank Ordered List

  

| SYMBOL | TITLE | RANK IN GENE LIST | RANK METRIC SCORE | RUNNING ES | CORE ENRICHMENT || 1 | GNRHR | na | 24 | 0.851 | 0.0839 | Yes |
| 2 | PLCB2 | na | 134 | 0.619 | 0.1424 | Yes |
| 3 | CACNA1F | na | 1554 | 0.333 | 0.1345 | Yes |
| 4 | GNRH1 | na | 2796 | 0.250 | 0.1235 | Yes |
| 5 | CALML6 | na | 3001 | 0.240 | 0.1415 | Yes |
| 6 | CAMK2B | na | 3930 | 0.203 | 0.1349 | Yes |
| 7 | PLA2G4E | na | 4751 | 0.174 | 0.1285 | Yes |
| 8 | ADCY3 | na | 5276 | 0.158 | 0.1291 | Yes |
| 9 | ADCY2 | na | 5416 | 0.154 | 0.1404 | Yes |
| 10 | ADCY9 | na | 5439 | 0.153 | 0.1550 | Yes |
| 11 | ADCY7 | na | 5559 | 0.149 | 0.1664 | Yes |
| 12 | ELK1 | na | 5841 | 0.141 | 0.1723 | Yes |
| 13 | SOS1 | na | 6194 | 0.132 | 0.1753 | Yes |
| 14 | PLA2G3 | na | 6310 | 0.129 | 0.1848 | Yes |
| 15 | CAMK2A | na | 6879 | 0.114 | 0.1798 | No |
| 16 | ADCY1 | na | 7855 | 0.095 | 0.1611 | No |
| 17 | MMP14 | na | 8454 | 0.084 | 0.1522 | No |
| 18 | PLA2G12B | na | 9215 | 0.072 | 0.1373 | No |
| 19 | PRKX | na | 9610 | 0.066 | 0.1325 | No |
| 20 | ITPR2 | na | 9655 | 0.065 | 0.1377 | No |
| 21 | SOS2 | na | 9955 | 0.061 | 0.1351 | No |
| 22 | MAP3K3 | na | 10136 | 0.059 | 0.1357 | No |
| 23 | ITPR1 | na | 10204 | 0.058 | 0.1395 | No |
| 24 | PLCB3 | na | 10228 | 0.057 | 0.1445 | No |
| 25 | ADCY4 | na | 10290 | 0.056 | 0.1484 | No |
| 26 | ADCY6 | na | 10456 | 0.054 | 0.1490 | No |
| 27 | PRKCA | na | 10617 | 0.052 | 0.1496 | No |
| 28 | KRAS | na | 10630 | 0.052 | 0.1544 | No |
| 29 | EGFR | na | 11115 | 0.046 | 0.1451 | No |
| 30 | MAPK9 | na | 11167 | 0.046 | 0.1481 | No |
| 31 | CAMK2G | na | 11259 | 0.045 | 0.1499 | No |
| 32 | MAPK11 | na | 11276 | 0.044 | 0.1539 | No |
| 33 | MAPK7 | na | 11510 | 0.041 | 0.1513 | No |
| 34 | MAP3K4 | na | 12080 | 0.035 | 0.1384 | No |
| 35 | PLD2 | na | 12343 | 0.033 | 0.1341 | No |
| 36 | MAP3K1 | na | 12394 | 0.032 | 0.1358 | No |
| 37 | MAPK12 | na | 13078 | 0.026 | 0.1187 | No |
| 38 | JUN | na | 13319 | 0.024 | 0.1142 | No |
| 39 | PLA2G2C | na | 13424 | 0.024 | 0.1136 | No |
| 40 | JMJD7-PLA2G4B | na | 14509 | 0.015 | 0.0838 | No |
| 41 | CALM2 | na | 14606 | 0.014 | 0.0824 | No |
| 42 | MAP2K7 | na | 14659 | 0.014 | 0.0823 | No |
| 43 | ATF4 | na | 14783 | 0.013 | 0.0800 | No |
| 44 | PLD1 | na | 14860 | 0.012 | 0.0790 | No |
| 45 | MAPK10 | na | 15299 | 0.009 | 0.0672 | No |
| 46 | GNA11 | na | 15711 | 0.006 | 0.0559 | No |
| 47 | GNAS | na | 16257 | 0.002 | 0.0404 | No |
| 48 | ADCY5 | na | 16504 | 0.000 | 0.0333 | No |
| 49 | PRKCD | na | 16865 | -0.002 | 0.0232 | No |
| 50 | MAP2K2 | na | 16997 | -0.003 | 0.0197 | No |
| 51 | CALML5 | na | 17116 | -0.004 | 0.0167 | No |
| 52 | LHB | na | 17219 | -0.005 | 0.0142 | No |
| 53 | PLA2G4A | na | 17245 | -0.005 | 0.0140 | No |
| 54 | PTK2B | na | 17352 | -0.006 | 0.0115 | No |
| 55 | MAPK14 | na | 17567 | -0.007 | 0.0060 | No |
| 56 | PLA2G6 | na | 17692 | -0.008 | 0.0033 | No |
| 57 | MAP2K4 | na | 17706 | -0.008 | 0.0037 | No |
| 58 | PLA2G12A | na | 17822 | -0.009 | 0.0013 | No |
| 59 | GNAQ | na | 17828 | -0.009 | 0.0021 | No |
| 60 | ITPR3 | na | 18064 | -0.011 | -0.0037 | No |
| 61 | PLCB4 | na | 18133 | -0.011 | -0.0045 | No |
| 62 | CDC42 | na | 18361 | -0.013 | -0.0098 | No |
| 63 | HRAS | na | 18435 | -0.013 | -0.0106 | No |
| 64 | CALML3 | na | 18646 | -0.015 | -0.0152 | No |
| 65 | CALM1 | na | 18762 | -0.015 | -0.0170 | No |
| 66 | PRKACB | na | 18969 | -0.017 | -0.0213 | No |
| 67 | CGA | na | 19555 | -0.021 | -0.0361 | No |
| 68 | CACNA1D | na | 19640 | -0.021 | -0.0364 | No |
| 69 | PLA2G2E | na | 19951 | -0.023 | -0.0430 | No |
| 70 | MAP2K3 | na | 20612 | -0.028 | -0.0593 | No |
| 71 | MAPK13 | na | 20970 | -0.031 | -0.0665 | No |
| 72 | MAPK3 | na | 22614 | -0.046 | -0.1093 | No |
| 73 | CALM3 | na | 22971 | -0.050 | -0.1147 | No |
| 74 | GRB2 | na | 23043 | -0.051 | -0.1117 | No |
| 75 | RAF1 | na | 23336 | -0.054 | -0.1148 | No |
| 76 | PRKACA | na | 23665 | -0.058 | -0.1185 | No |
| 77 | PLA2G10 | na | 23788 | -0.060 | -0.1161 | No |
| 78 | SRC | na | 24048 | -0.063 | -0.1173 | No |
| 79 | MAPK8 | na | 24525 | -0.070 | -0.1241 | No |
| 80 | MAPK1 | na | 24626 | -0.071 | -0.1199 | No |
| 81 | CACNA1C | na | 25293 | -0.080 | -0.1312 | No |
| 82 | GNRH2 | na | 25357 | -0.081 | -0.1250 | No |
| 83 | PRKACG | na | 25752 | -0.087 | -0.1277 | No |
| 84 | HBEGF | na | 25935 | -0.089 | -0.1241 | No |
| 85 | ADCY8 | na | 25957 | -0.090 | -0.1158 | No |
| 86 | MAP3K2 | na | 26106 | -0.092 | -0.1109 | No |
| 87 | NRAS | na | 27790 | -0.126 | -0.1471 | No |
| 88 | PLA2G2D | na | 27985 | -0.130 | -0.1397 | No |
| 89 | MAP2K6 | na | 28948 | -0.155 | -0.1520 | No |
| 90 | MMP2 | na | 30057 | -0.190 | -0.1652 | No |
| 91 | PLA2G2F | na | 30623 | -0.208 | -0.1608 | No |
| 92 | MAP2K1 | na | 31070 | -0.223 | -0.1515 | No |
| 93 | PLA2G5 | na | 32093 | -0.264 | -0.1548 | No |
| 94 | CAMK2D | na | 32170 | -0.268 | -0.1303 | No |
| 95 | PLCB1 | na | 32251 | -0.272 | -0.1056 | No |
| 96 | PLA2G2A | na | 32310 | -0.275 | -0.0799 | No |
| 97 | FSHB | na | 33243 | -0.340 | -0.0730 | No |
| 98 | PLA2G1B | na | 33526 | -0.362 | -0.0451 | No |
| 99 | PRKCB | na | 33863 | -0.399 | -0.0151 | No |
| 100 | CACNA1S | na | 33887 | -0.403 | 0.0243 | No |
Table: GSEA details [plain text format]

  

Fig 2: KEGG\_GNRH\_SIGNALING\_PATHWAY      
 Blue-Pink O' Gram in the Space of the Analyzed GeneSet

  

Fig 3: KEGG\_GNRH\_SIGNALING\_PATHWAY: Random ES distribution      
 Gene set null distribution of ES for **KEGG\_GNRH\_SIGNALING\_PATHWAY**
